# The Cultivation of Trypanosoma from the Leishman-Donovan Body

**Published:** 1905-01-14

**Authors:** 


### THE CULTIVATION OF TRYPANOSOMA FROM THE LEISHMAN-DONOVAN BODY.

FOLLOWING up Captain Rogers's announcement that he had observed the development of trypanosomes from the Leishman-Donovan body, Mr. G. C. Chatterjee, in an illustrated contribution to last week's *Lancet*, verifies this observation. Working under Captain Rogers, and adopting his method, Mr. Chatterjee has been able to watch the process throughout. Some blood was taken from the spleen of a patient who was suffering with fever, enlarged spleen, and anæmia. Three members of the patient's family had recently died from fever with enlarged spleen, and œdema of the legs. The blood was placed in a test-tube containing a small quantity of 5 per cent. sodium citrate solution which had previously been sterilised. A stained slide preparation of the blood thus diluted made on the first day showed a good number of distinct Leishman-Donovan bodies, from three to four being found in a single field. On the next day a stained specimen showed a great increase in the number of the parasites, so that as many as 200 or even 500 could be counted in a single field; the parasites now showed increased development, and were distinctly larger than before, and were actively dividing. No flagellate forms were found on this day. On the third day all the stages of growth could be seen from a simple Leishman-Donovan body to an adult trypanosoma-like body. The fully-developed parasites showed certain characters which led Mr. Chatterjee to suggest that they are more closely allied to the class of trypanopis than to the trypanosomata. The matter is of importance because it bears upon a new field of inquiry in tropical medicine.

